# Appropriateness of Antibiotic Prescriptions Written for Dental Prophylaxis Within a Regional Veterans Affairs Healthcare System Based on American Dental Association and American Academy of Orthopaedic Surgeons Guidance

**DOI:** 10.1093/ofid/ofaf432

**Published:** 2025-07-24

**Authors:** David B Portman, Jeffrey B Doyon, Deanna J Buehrle

**Affiliations:** Department of Pharmacy, Veterans Affairs Butler Healthcare System, Butler, Pennsylvania, USA; Department of Medicine, Veterans Affairs Philadelphia Healthcare System, Philadelphia, Pennsylvania, USA; Department of Medicine, Veterans Affairs Pittsburgh Healthcare System, Pittsburgh, Pennsylvania, USA

**Keywords:** antibiotic prophylaxis, dental, orthopedic, prescribing, stewardship

## Abstract

**Background:**

We determined the prevalence of inappropriate dental prophylaxis antibiotic prescriptions written by providers within a Veterans Integrated Service Network based on American Dental Association (ADA) and American Academy of Orthopaedic Surgeons (AAOS) guidance.

**Methods:**

We conducted a retrospective cohort study of antibiotic prescriptions written for dental prophylaxis from 1 May 2023 through 30 April 2024. Prescriptions were defined as indicated if the patient met ADA guidance criteria. A subset of prescriptions written for patients with orthopedic implants was assessed for appropriateness when applying the 2017 and 2024 AAOS guidelines.

**Results:**

Of 622 prescriptions, 53%, 37%, and 10% were written due to underlying orthopedic, cardiac, and other conditions, respectively. Dentists prescribed 51% of prescriptions, followed by primary care providers (35%) and orthopedic specialists (9%). Overall, 67% prescriptions were not indicated. When considering indication, agent, and dose, 72% of prescriptions were inappropriate. Prescriptions written due to cardiac conditions were more likely to be indicated than prescriptions written due to orthopedic conditions (*P* < .0001). Prescriptions written by dentists were more likely to be indicated than prescriptions written by primary care providers (*P* = .004) or orthopedic specialists (*P* < .0001). When applying 2017 AAOS guidelines to prescriptions written due to orthopedic conditions (n = 330), 0%, 8%, and 92% were considered “appropriate,” “maybe appropriate,” and “rarely appropriate,” respectively. When applying 2024 AAOS guidelines, 100% were not indicated.

**Conclusions:**

Our results suggest substantial antibiotic overuse and lack of adherence with ADA and AAOS recommendations, particularly among patients with orthopedic implants. These results highlight the need for multifaceted stewardship interventions targeting not only dentists, but all types of providers.

## Teaser

A retrospective cohort study of antibiotic prescribing by all provider types for dental prophylaxis within a regional Veterans Affairs system found substantial lack of adherence with ADA and AAOS recommendations, particularly among patients with orthopedic implants.

Approximately 10% of all outpatient antibiotic prescriptions in the United States (US) are written by dentists [1]. Dentists are among the top antibiotic prescribers, after only primary care physicians, physician assistants, and nurse practitioners, writing 25.2 million antibiotic prescriptions during the 2022 calendar year [1]. One of the most common indications for antibiotics prescribed by dentists is prophylaxis of bacterial infections [2]. There are limited data on how often non-dentists prescribe dental antibiotic prophylaxis [3].

The American Dental Association (ADA) and the American Academy of Orthopaedic Surgeons (AAOS) have published guidance that address antibiotic prophylaxis in patients undergoing dental procedures [4–6]. The ADA recommends antibiotic prophylaxis for only a small number of patients with specific cardiac conditions (citing clinical practice guidelines published by the American Heart Association in 2021) who are undergoing invasive dental procedures that include gingival manipulation or mucosal incision [4, 7]. In January 2015, the ADA published guidelines specifically addressing patients with prosthetic joint implants that recommended against prophylactic antibiotics prior to dental procedures [5]. The AAOS guidelines address only patients with orthopedic implants. In 2017, AAOS published appropriate use criteria for managing patients with orthopedic implants who are undergoing dental procedures [6]. These guidelines differed from the ADA guidelines and recommended antibiotic prophylaxis for some patients with orthopedic implants who met certain conditions. In November 2024, however, the AAOS revised its guidelines, citing updated literature, to state that routine use of prophylactic antibiotics prior to dental procedures may not reduce risk of prosthetic joint infection among patients with prosthetic joints [8]. Following the revision of the AAOS guidelines, the current guidance from AAOS and ADA regarding dental antibiotic prophylaxis aligns. Thus, only a small subset of patients with certain cardiac conditions undergoing dental procedures meet criteria for antibiotic prophylaxis under current guidance.

Despite these narrow criteria, a study reporting on US patients with commercial dental insurance from 2016 through 2018 found that 77% to 78.5% of antibiotic prophylaxis was unnecessary [8]. History of a prosthetic joint had one of the strongest associations with unnecessary prophylaxis. This has led to calls for antimicrobial stewardship interventions in dental practices [1, 9]. To our knowledge, there are no published studies evaluating the change in antibiotic appropriateness for dental prophylaxis when applying the old AAOS guidelines (2017) and the new AAOS guidelines (2024). The objective of this study was to determine the prevalence of inappropriate antibiotic prescriptions for dental prophylaxis based on ADA recommendations written by all provider types (not only dentists) within a regional Veterans Integrated Services Network (VISN) and to describe appropriateness when applying AAOS guidelines.

## METHODS

We conducted a retrospective cohort study between 1 May 2023 and 30 April 2024 of Veterans Affairs (VA) patients receiving care within VISN 4 (which is comprised of 9 medical centers, each with their own dental clinic, and 46 outpatient clinics located in Pennsylvania, Delaware, and parts of Ohio, West Virginia, New York, and New Jersey) who received an antibiotic prescription for dental prophylaxis. As the data presented here were gathered as part of quality improvement activities, VA Institutional Review Board approval was waived.

### Study Population

All outpatient prescriptions of oral systemic antibiotics dispensed within the VISN from VA pharmacies during the timeframe were catalogued from pharmacy dispensing data. To narrow this list to only prescriptions for dental indications, we included only prescriptions that contained the term “dental” within the instructions or indication field. The charts of the patients receiving each antibiotic prescription were reviewed by 1 of the investigators (D. B. P.) to determine if it was prescribed for the indication of dental prophylaxis. Any prescription that did not meet this criterion was excluded. A random one-third (approximately 33%) of prescriptions were selected to undergo comprehensive chart review by an antimicrobial stewardship pharmacist or physician at the VA facility from which the prescription was dispensed. Reviewers received a data collection instruction manual and a standardized data collection sheet, as well as verbal instruction on the process to limit interreviewer variability.

### Study Definitions

An antibiotic prescription was defined as indicated if the patient had a qualifying cardiac condition as defined in American Heart Association (AHA) guidelines, which are cited in ADA guidance [4, 7]. Qualifying cardiac conditions were any of the following: prosthetic cardiac valve or valve repair with prosthetic valve material, durable mechanical circulatory support device, a history of infective endocarditis, certain types of congenital heart disease as defined in the AHA guidelines, receipt of a cardiac transplant with valve regurgitation attributable to a structurally abnormal valve, or a left atrial appendage occlusion device within the previous 6 months [7 ].

Among indicated prescriptions, a guideline-concordant agent was defined as amoxicillin unless the patient had a penicillin class allergy. In the presence of a penicillin class allergy, cephalexin was defined as a guideline-concordant agent except in individuals with a history of an immunoglobulin E mediated/type I allergic reaction (eg, anaphylaxis, angioedema, urticaria, wheezing/shortness of breath) in which case either azithromycin or doxycycline was guideline-concordant. A guideline-concordant dose was defined as a one-time dose of amoxicillin 2000 mg, cephalexin 2000 mg, azithromycin 500 mg, or doxycycline 100 mg [7]. A prescription was deemed appropriate if it was indicated and a guideline-concordant agent and dose were used.

As a secondary measure, we investigated appropriateness of prophylaxis among patients with orthopedic implants when applying the 2017 and 2024 AAOS guidelines [6 , 8 ]. The 2017 AAOS guidelines recommended risk-stratifying patients with orthopedic implants into 1 of 3 categories (antibiotics are “appropriate,” antibiotics “may be appropriate,” or antibiotics are “rarely appropriate”) based on patient-specific risk factors [6 ]. We used the AAOS Care Decision Tree to stratify patients with orthopedic implants into 1 of these categories [10]. Patient specific risk factors included immunocompromised status (defined within AAOS Care Decision Tree), history of prosthetic joint infection, time since operation, and A1C or blood glucose status. The 2024 AAOS guidelines were published after the conclusion of our data collection. We applied the 2024 AAOS guidelines to our existing data set to identify the baseline appropriateness of prescriptions when applying these new guidelines, and to assist with identifying target areas for future interventions. All prescriptions written for patients with orthopedic implants (without qualifying cardiac conditions) were considered not indicated and inappropriate under the 2024 AAOS guidelines as they do not recommend routine prophylaxis [8 ].

### Data Collection and Statistical Analysis

Prescription data included date of prescription, drug, dose, frequency, quantity, number of refills, prescriber, and prescriber specialty. Descriptive characteristics such as demographic data, antibiotic allergy information, dental procedure category, and presence and status of applicable comorbid conditions (prosthetic joints, cardiac conditions, immunocompromised status, and diabetes) were gathered from the electronic medical record. Comparisons involving categorical variables were made using Fisher exact tests.

## RESULTS

During the study period, 55 811 antibiotic prescriptions were dispensed. Among these, 1806 prescriptions were written for dental prophylaxis. Chart review was conducted on 34% (622/1806) of the prescriptions written for dental prophylaxis.

The 622 prescriptions were written for 536 unique patients; 14% (86/622) of patients were given >1 prescription. The median age of the cohort was 74 years (range, 36–97 years), 93% (500/536) were male, and 88% (469/536) were White. Nineteen percent (101/536) of patients had at least 1 antibiotic allergy label. Of patients with an antibiotic allergy label, 63% (63/101) had an allergy label for a β-lactam antibiotic. Of the β-lactam allergy labels, 56% (35/63) were for non-anaphylactic reactions.

The characteristics of the antibiotic prescriptions are presented in Table 1. Of the 622 prescriptions reviewed, 45% (279/622) were written for a quantity that would cover 1 course of prophylaxis (eg, single course prior to 1 dental procedure); 8% (48/622) were written for a quantity that would cover 2 courses of prophylaxis, 14% (87/622) were written for a quantity that would cover 3 courses of prophylaxis, and 33% (208/622) were written for a quantity that would cover 4 or more courses of prophylaxis. Forty-eight percent (296/622) of prescriptions had at least 1 refill prescribed in addition to the original fill; 11% (69/622) had ≥4 refills in addition to the original fill prescribed. There were 3309 total courses (original courses plus courses prescribed in refills) of prophylaxis prescribed for 536 patients (mean of 6.2 courses per patient). The most prescribed agents were amoxicillin (82% [510/622]), clindamycin (8% [48/622]), azithromycin (4% [26/622]), and cephalexin (3% [21/622]). There were 218 unique prescribers identified. Dentists prescribed 51% (319/622) of prescriptions, followed by primary care providers (35% [215/622]), orthopedic specialists (9% [56/622]), cardiology specialists (4% [26/622]), and other providers (1% [6/622]).

**Table 1.** Characteristics of Antibiotic Prescriptions (N = 622)

Characteristic	No. (%)
Prescription type	
New	472 (76)
Renewal	150 (24)
Antibiotic	
Amoxicillin	510 (82)
Clindamycin	48 (8)
Azithromycin	26 (4)
Cephalexin	21 (3)
Doxycycline	13 (2)
Penicillin VK	3 (<1)
Amoxicillin/clavulanate	1 (<1)
Dosing frequency	
Once	611 (98)
Multiple	11 (2)
No. of courses dispensed^a^	
1	279 (45)
2	48 (8)
3	87 (14)
4	119 (19)
≥5	89 (14)
No. of refills	
0	326 (52)
1	67 (11)
2	93 (15)
3	67 (11)
≥4	69 (11)
Prescription pick-up method	
Mail	456 (73)
Window	166 (27)
Total courses prescribed^b^	
≤2	186 (30)
3–4	177 (29)
5–6	105 (17)
≥7	154 (25)
Prescriber specialty	
Dental	319 (51)
Primary care	215 (35)
Orthopedics	56 (9)
Cardiology	26 (4)
Other	6 (1)
Prescriber type	
Dentist	319 (51)
MD/DO	155 (25)
CRNP	84 (14)
PA	63 (10)
Unknown	1 (<1)

Abbreviations: CRNP, Certified Registered Nurse Practitioner; DO, Doctor of Osteopathic Medicine; MD, Doctor of Medicine; PA, Physician Assistant.

^a^A course of antibiotics was defined as the quantity of medication that would be required to cover 1 dental procedure. For example, a prescription of 8 tablets of amoxicillin with the directions “take 4 tablets once prior to dental procedure” counted as 2 courses.

^b^The number of courses covered by the original prescription plus the number of courses prescribed in refills.

Fifty-three percent (330/622) of the prescriptions were written due to underlying orthopedic conditions, 37% (231/622) were written due to cardiac conditions, 4% (26/622) were written due to immunosuppressive conditions, 5% (33/622) were written for unknown conditions, and <1% (2/622) were written due to Agent Orange exposure or use of hemodialysis. One hundred percent (330/330), 10% (24/231), 100% (26/26), and 100% (35/35) of prescriptions for orthopedic, cardiac, immunosuppressive, and other or unknown conditions were not indicated, respectively (Table 2). Overall, 67% (415/622) of prescriptions were not indicated. Among prescriptions that were indicated, 13% (27/207) were for a guideline-discordant agent. Among prescriptions that were indicated and written for a guideline-concordant agent, 2% (3/180) were for a guideline-discordant dose. Therefore, 28% (177/622) of prescriptions were appropriate when considering whether the prescription was indicated, the agent, and the dose. Prescriptions written for cardiac conditions were more likely to be indicated than prescriptions written for orthopedic conditions (90% [207/231] vs 0% [0/330], respectively, *P* < .0001) or immunosuppressive conditions (90% [207/231] vs 0% [0/26], respectively, *P* < .001). Prescriptions written by dentists were more likely to be indicated than prescriptions written by primary care providers (39% [126/319] vs 27% [59/215], respectively, *P* = .004) or orthopedic specialists (39% [126/319] vs 0% [0/56], respectively, *P* < .0001).

**Table 2.** Factors Associated With Unnecessary Dental Prophylaxis Antibiotic Prescriptions

Factor	Total (N = 622)	Antibiotic Prescription Indicated^a^ (n = 207)	Antibiotic Prescription Not Indicated (n = 415)	Percent Unnecessary Prophylaxis
Underlying condition, No. (%)				
Orthopedic	330 (53)	0 (0)	330 (80)	100
Cardiac	231 (37)	207 (100)	24 (6)	10.4
Immunosuppressive	26 (4)	0 (0)	26 (6)	100
Unknown	33 (5)	0 (0)	33 (8)	100
Agent Orange exposure	1 (<1)	0 (0)	1 (<1)	100
Hemodialysis	1 (<1)	0 (0)	1 (<1)	100
Prescriber specialty, No. (%)				
Dental	319 (51)	126 (61)	193 (46)	60.5
Primary care	215 (35)	59 (28)	156 (38)	72.6
Orthopedic	56 (9)	0 (0)	56 (14)	100
Cardiology	26 (4)	22 (11)	4 (1)	15
Nephrology	3 (<1)	0 (0)	3 (1)	100
Gastroenterology/hepatology	2 (<1)	0 (0)	2 (1)	100
Other surgical	1 (<1)	0 (0)	1 (<1)	100

^a^An antibiotic prescription was defined as indicated if the patient had a qualifying cardiac condition as defined in American Heart Association guidelines, which are cited by the American Dental Association.

Orthopedic indications were listed for 53% (330/622) of prescriptions. The median number of days elapsed from the orthopedic procedure to the antibiotic prophylaxis prescription was 1114 days (range, −91 to 9583 days), with 227 (68.8%) prescriptions being written >1 year after the procedure. Of the prescriptions written for orthopedic indications, 55.8% (184/330), 39.1% (129/330), and 17% (56/330) were written greater than 2 years, 5 years, and 10 years, respectively, after the procedure date. Using the 2017 AAOS guidelines, 0% (0/330) of prescriptions were considered “appropriate,” 8% (25/330) were considered “maybe appropriate,” and 92% (305/330) were considered “rarely appropriate.” When applying the 2024 AAOS guidelines, 100% (330/330) of these prescriptions would not be indicated and thus inappropriate.

## DISCUSSION

To our knowledge, this is the first study to describe a comprehensive estimate of inappropriate antibiotic prescribing for dental prophylaxis based on both ADA and AAOS recommendations written by all provider types across a large, multi-hospital system. Our study was unique in describing all prescriptions written by any type of provider (not only dentists) for dental prophylaxis, conducting chart review for determination of indications and appropriateness, and describing a secondary measure of appropriateness based on the AAOS guidelines among patients with orthopedic conditions. Indeed, we found that prescriptions written by dentists were more likely to be indicated than those written by primary care providers or orthopedic specialists. In addition to analyzing whether an antibiotic is indicated, we also analyzed antibiotic agent and dose, along with number of courses and refills prescribed.

We observed substantial antibiotic overuse and a lack of adherence with ADA and AAOS guidance. First, we found that two-thirds of antibiotic prescriptions written for dental prophylaxis were not indicated. The predominant indication for prophylaxis cited among our cohort was underlying orthopedic conditions. When applying ADA guidance, 0% of prescriptions written for patients with orthopedic conditions were indicated whereas 90% of prescriptions written for patients with cardiac conditions were indicated. Second, even when using the 2017 AAOS guidelines Care Decision Tree to determine appropriate use among patients with orthopedic conditions, 0% of the prescriptions written met criteria for “appropriate” use and only 8% were consider “maybe appropriate.” Last, patients vastly received a larger supply of antibiotics and more antibiotic refills than necessary. Over half of prescriptions were written for a quantity that would cover more than 1 course of prophylaxis and nearly half of prescriptions were written with refills. While some of these refills may have been written for patient convenience, the excess antibiotic supply at home allows for potential inappropriate antibiotic use.

One of the most unique aspects of our study was that we gathered patient specific data to stratify prescriptions written for patients with orthopedic implants using the AAOS appropriate use criteria published in 2017. When we applied these guidelines to prescriptions written for orthopedic conditions, the vast majority (92%) were still considered “rarely appropriate” and 0% were considered “appropriate.” If applying the newly released 2024 AAOS guidelines, 0% would be considered appropriate. Thus, even when applying the AAOS guidelines, most of the dental antibiotic prophylaxis written due to orthopedic conditions is not indicated. We are not aware of any other studies that have applied the 2017 or 2024 AAOS guidelines to determine antibiotic prophylaxis appropriateness among cohorts of patients.

Like our study, others have reported starkly high rates of inappropriate antibiotic prescribing for dental prophylaxis. Investigators reviewing dental, medical, and pharmacy claims from a sample of 8 million persons in the US with commercial insurance found that over 77% of antibiotic prophylaxis was unnecessary from 2016 through 2018 [11]. A similar study, conducted using data from 2011 to 2015, described a comparable rate of inappropriate prescribing (about 81%), which suggests that the problem did not improve over these years [2]. In both studies, prosthetic joint devices were associated with unnecessary prophylaxis, like our findings. Within the VA, there was a study conducted of dental prophylaxis prescriptions written prior to dental visits from 2015 to 2019 that found every 5 of 6 prescriptions were inconsistent with AHA guidelines [12]. While the findings of the study were like ours, it differed from ours because it only included prescriptions written by dentists. Additionally, all these studies used diagnosis codes to determine underlying patient characteristics whereas our study used chart review to determine the indication that the provider used to justify antibiotic prescribing. These studies also only examined appropriateness by qualifying cardiac conditions and did not consider AAOS guidelines.

Taken together, our study has important implications for designing antimicrobial stewardship interventions focused on dental prescribing. Educating providers about appropriate use of prophylaxis among patients with orthopedic implants is a top priority. Most prescriptions for antimicrobial prophylaxis came from dentists, primary care providers, and orthopedic specialists, suggesting that any stewardship intervention should be multidisciplinary. Key stewardship interventions include ensuring appropriate indications for prophylaxis, using appropriate agents and doses, and limiting the number of courses and refills prescribed, as well as ongoing reassessment and shared decision-making with patients on the need for prophylaxis. Indeed, an academic detailing intervention conducted at 1 of our centers improved prescribing appropriateness of antibiotic prophylaxis prior to dental procedures from 18% to 90% (*P* < .001) [13]. Other types of stewardship interventions that have been conducted in the outpatient setting include provider education, provider feedback, implementation of guidelines, delayed prescribing, restrictive policies, and computerized decision support [14 ]. However, dental stewardship interventions are not well described. A recent meta-analysis of dental antibiotic stewardship interventions found that multifaceted interventions involving in-person education, audit and feedback, and behavioral change messaging improved antibiotic prescribing by dentists [15 ]. There was no reduction in prescribing using a guideline posting intervention. There was also no benefit to using peer comparison when compared with standard audit and feedback. However, in this meta-analysis, only 1 study included high-certainty evidence. Given the number of distinct prescriber groups identified in this study, tailored interventions may be key to reducing inappropriate antibiotic prescribing. Interventions will need to address potential reasons why inappropriate antibiotic prescribing is so high for dental prophylaxis. These reasons may include lack of awareness of guidelines or recent guideline changes, pressure from patients given long-term prior use of prophylaxis, and provider fears related to infections if they do not prescribe antibiotics [16].

Limitations of this study include its retrospective design and inclusion of only 9 VA hospitals in 1 region. Therefore, the results may not be generalizable to other regions or to private healthcare settings. Decisions regarding indications and appropriateness of antibiotic prescribing assume accurate chart documentation. As such, it is possible that some prescriptions were misclassified due to incomplete documentation. Additionally, we reviewed a subset of randomly chosen prescriptions. Since we used prescriptions as the starting point (rather than dental visits), it is possible that even more prescriptions were inappropriate if the dental procedure did not involve gingival manipulation or mucosal incision. Last, we included only prescriptions for which the word “dental” was written within the prescription. It is possible that our results could differ if we analyzed a larger number of prescriptions and not only prescriptions that contained the word “dental.” However, our data are like other studies that have reported appropriateness of antibiotic prescribing for dental prophylaxis.

In conclusion, more than 65% of antibiotic prescriptions written for dental prophylaxis were not indicated and there is opportunity to improve in 72% of prescriptions when considering antibiotic choice and dose. While antibiotic prophylaxis is only recommended in patients with specific cardiac conditions, most prescriptions were written due to orthopedic conditions. New AAOS guidelines were published in 2024 that state routine use of prophylaxis may not reduce infection risk among patients with prosthetic joints. It is possible that these guidelines could help to improve practice. However, antimicrobial stewardship interventions targeting dental prescribing are imperative.

## Notes

***Collaborators in the VISN 4 Antimicrobial Stewardship Committee.*** James E. Van Zandt VA Medical Center: Chelsey Chambers, PharmD, Sung Mo, PharmD, Julia Wasser, PharmD. Coatesville VA Medical Center: Davy Peou, PharmD. Corporal Michael J. Crescenz VA Medical Center: Melissa White, PharmD, Jonathan Burns, PharmD. Erie VA Medical Center: Amy Newel, PharmD. Lebanon VA Medical Center: Elizabeth Depew, PharmD. VA Pittsburgh Healthcare System: Abigail Beck, PharmD. Wilkes-Barre VA Medical Center: Carina Mackarey, PharmD, Mia Barbieri, Brooke Henninger, Gabriela Smicherko. Wilmington VA Medical Center: Anna Drapkin, PharmD, Stefania DelPrete.
